# Effects of Atrioventricular Interval Modulation Therapy in Subjects With Hypertension and Diastolic Dysfunction

**DOI:** 10.1016/j.jacadv.2025.101896

**Published:** 2025-07-23

**Authors:** Marat Fudim, Yuval Mika, Avi Fischer, Steven J. Evans, Ziv Belsky, Karl-Heinz Kuck

**Affiliations:** a Department of Medicine, Duke University Medical Center, Durham, North Carolina, USA; b Duke Clinical Research Institute, Durham, North Carolina, USA; c Orchestra BioMed, Inc, New Hope, Pennsylvania, USA; d Department of Cardiology, University Heart Center Lübeck, University Hospital Schleswig-Holstein, Lübeck, Germany

**Keywords:** atrioventricular interval modulation therapy, diastolic dysfunction, hypertension

Long-standing hypertension leads to cardiac structural and functional changes that frequently result in left ventricular (LV) diastolic dysfunction (DD). The progression of DD is directly linked to the development of heart failure (HF) even in the presence of preserved ejection fraction.^1^ Globally, more than one million patients are implanted with pacemakers annually, with over 70% of these patients suffering from hypertension. The high prevalence of hypertension is primarily attributable to the advanced age of pacemaker recipients along with the elevated incidence of other significant cardiovascular diseases. Atrioventricular interval modulation (AVIM) therapy, also called cardiac neuromodulation therapy, employs a sequence of variably timed short and longer atrioventricular intervals during dual-chamber pacing to yield sustained reductions of systolic blood pressure (SBP) in patients with hypertension. Clinical studies have demonstrated that AVIM therapy immediately, substantially, and persistently reduces blood pressure (BP) in patients indicated for a dual-chamber pacemaker who also have uncontrolled hypertension.^2^^,^^3^ LV pressure-volume analyses establish that AVIM therapy reduces ventricular filling (preload) and decreases total peripheral resistance (afterload) while modulating the autonomic nervous system and therefore has the potential to improve diastolic function. Accordingly, we evaluated whether the presence of DD impacts the efficacy of AVIM therapy to reduce BP and if AVIM therapy improves DD.

**What is the clinical question being addressed?**

Is AVIM therapy delivered during dual-chamber pacing associated with favorable changes in patients with hypertension and diastolic dysfunction?

**What is the main finding?**

These data demonstrate that AVIM therapy is associated with a comparable reduction in systolic blood pressure in patients with and without diastolic dysfunction. In addition, in patients with diastolic dysfunction, AVIM therapy leads to favorable echocardiographic changes consistent with improved myocardial relaxation and diastolic compliance.

## Methods

This is a secondary analysis of the MODERATO II study (NCT02837445), a prospective, multicenter, randomized, double-blind trial evaluating the efficacy and safety of AVIM in patients with an indication for a dual-chamber pacemaker and uncontrolled hypertension despite medication. The study required patients to have a LV ejection fraction >50% and the absence of HF symptoms.^3^ Patients had to be in sinus rhythm at the time of enrollment. Patients with persistent or permanent atrial fibrillation (AF) were excluded from the study, and patients with paroxysmal AF were included only if the AF burden was <25%. Eligible patients had daytime ambulatory SBP ≥130 mm Hg and office SBP ≥140 mm Hg despite taking ≥1 antihypertensive medication, and an indication for a dual-chamber pacemaker. The study received ethics approval at each respective trial site, all patients provided informed consent, and were implanted with the Moderato device (BackBeat Medical, an Orchestra BioMed company) prior to being randomly assigned to a control group or a treatment group receiving AVIM therapy for 6 months (primary endpoint). The study did not require stratification based on indication for pacemaker implant or percent pacing.

All patients with echocardiographic images suitable for the evaluation of DD were included in this analysis. Echocardiograms were assessed to determine the presence of DD according to American Society of Echocardiography guideline criteria (≥2 of the following): 1) average E/e' >14 or septal e' <7 cm/s or lateral e'<10 cm/s or tricuspid regurgitation >2.8 m/s; 2) left atrial volume index >34 mL/m^2^; and 3) LV wall thickness >1.1 cm.^4^ Echocardiographic images were reviewed in a blinded fashion to treatment assignment and timing. The effects of 6 months of AVIM therapy on SBP were compared between patients with (DD+) and without DD (DD−) using 24-hour ambulatory and office measurements. In addition, the impact of 6 months of AVIM therapy on DD was compared between treatment and control groups. Descriptive statistics (means, medians, SDs, percentages, and CIs) were used to summarize the findings. Data are presented as mean ± SD and comparisons were made using paired 2-sided *t*-tests with *P* values <0.05 considered statistically significant. Bonferroni correction was applied to account for multiple comparisons of the echo data.

## Results

From the MODERATO II study cohort (n = 47), 36 patients had technically adequate echocardiographic images and 22 (12 treatment and 10 control) met criteria for DD+ while 14 subjects (11 treatment, 3 controls) did not meet criteria (DD−). Patients averaged 73.6 years of age, 64% were men, and there was a high prevalence of comorbidities, including diabetes mellitus (44%), coronary artery disease (44%), and history of atrial fibrillation (22%). The Moderato system was a first-time pacemaker implant for 61% of the patients and was a replacement device in 39%. The main indications for pacing were sick sinus syndrome and second-degree atrioventricular conduction block. Patients in the treatment group were paced virtually 100% of the time with average ventricular pacing of 99.8% ± 0.5%. The control group was programmed at the discretion of the physician based on underlying disease and indications. There were 2 pacemaker-dependent patients in the treatment arm and 1 in the control arm. Patients were taking a median of 3 antihypertensive medications.

The impact of AVIM therapy on cardiac function in the DD+ group is summarized in Table 1. In comparison to control, treatment with AVIM therapy was associated with increases in e' (from 5.9 ± 2.0-8.8 ± 3.4 cm/s; *P* < 0.01) consistent with an improvement in LV relaxation, and E (from 61.3 ± 15.9 to 89.3 ± 17.5; *P* < 0.01) consistent with improved passive filling despite reduced filling time (effect of AVIM therapy).

**Table 1:** Review of Echocardiographic Parameters From Baseline to 6 Months in the Atrioventricular Interval Modulation Therapy Group and the Sham-Control Group

	Treatment			Sham-Control		
	Baseline (N = 12)	6M	Difference Between 6M and Baseline	Baseline (N = 10)	6M	Difference Between 6M and Baseline
Left ventricular ejection fraction (%)	64.8 ± 6.8	61.8 ± 5.3	−3.0 ± 7.2	60.4 ± 4.1	59.7 ± 3.2	−0.57 ± 5.6
Left atrial size	41.1 ± 5.3	42.7 ± 5.6	1.6 ± 3.1	41.5 ± 4.9	43.3 ± 4.1	1.8 ± 2.2^a^
E	61.3 ± 15.9	89.3 ± 17.5	28.0 ± 18.6^a^	63.6 ± 20.6	65.3 ± 22.4	1.7 ± 14.3
e'	5.9 ± 2.0	8.8 ± 3.4	2.8 ± 2.9^a^	5.5 ± 2.0	6.5 ± 2.0	1.0 ± 3.1
E/e'	11.2 ± 4.1	11.1 ± 3.1	−0.08 ± 3.9	12.1 ± 3.9	10.9 ± 4.4	−1.2 ± 5.9

Values are mean ± SD.

a *P* < 0.05.

When comparing 6-month results based on group assignment during the study, ambulatory SBP was reduced in treatment DD+ patients (N = 12) by 8.3 ± 9.7 mm Hg (*P* < 0.01 vs baseline) compared to 13.9 ± 9.3 mm Hg (*P* < 0.01 vs baseline) in treatment DD− patients (N = 11, *P* = 0.08 between groups). Office SBP was reduced in treatment DD+ by 12.1 ± 12.8 mm Hg (*P* < 0.01 vs baseline) compared to 11.5 ± 12.7 mm Hg (*P* < 0.01 vs baseline) in treatment DD− (*P* = 0.93 between groups). In the control DD+ group (N = 10), ambulatory SBP was reduced by only 2.2 ± 9.8 mm Hg and office SBP was increased by 2.9 ± 26.4 mm Hg.

## Conclusions

AVIM therapy represents a novel device-based treatment aimed at reducing BP in patients with uncontrolled hypertension. AVIM therapy works by modulating the timing of atrioventricular conduction during dual-chamber pacing to reduce ventricular filling and preload, reducing intracardiac volumes and pressures. This mechanism of action might be particularly effective in patients with DD, where impaired ventricular relaxation and increased myocardial stiffness lead to elevated filling pressures and reduced cardiac efficiency.

Evidence from the MODERATO II study highlights AVIM therapy's efficacy in reducing BP, a critical determinant of DD. Elevated BP contributes to increased LV wall stress and stiffness, exacerbating the already impaired diastolic relaxation in these patients. By achieving sustained BP reductions, AVIM therapy lowers afterload and LV filling pressures, facilitating better myocardial relaxation and improving diastolic compliance. Whether these effects translate into improved LV function, including enhanced stroke volume and reduced myocardial strain, remains to be determined and could not be extracted from our data.

In addition to its BP-lowering effects, AVIM therapy could exert direct benefits on LV structure and function. By reducing chronic wall stress, AVIM therapy may promote reverse remodeling and prevent further progression of hypertensive heart disease. Improved hemodynamics may also enhance overall myocardial efficiency. Future trials will be important in determining whether the observed changes in SBP and DD improve health status and clinical outcomes in patients with HF with preserved ejection fraction.

Notably, in the present cohort of patients with uncontrolled hypertension and an indication for a pacemaker, 61% had echocardiographic evidence of DD. AVIM appeared to be effective in reducing BP irrespective of underlying DD and favorably affected DD during follow-up. The ongoing BACKBEAT (BradycArdia paCemaKer with AV interval modulation for Blood prEssure treAtmenT) trial (NCT06059638) aims to further characterize the safety and effectiveness of AVIM therapy in patients indicated for a dual-chamber pacemaker who also have uncontrolled hypertension.

## Funding support and author disclosures

Dr Kuck was an investigator in the MODERATO II trial. Drs Mika, Fischer, Evans, and Belsky are employees of Orchestra BioMed, Inc. Dr Fudim is a consultant to Orchestra BioMed, Inc. The study was sponsored by BackBeat Medical (an Orchestra BioMed company).
